# Orthodontic Need Among Adult Patients Seeking Oral Rehabilitation in Craiova

**DOI:** 10.3390/jcm15114312

**Published:** 2026-06-02

**Authors:** Amelia Smaranda Roșianu, Antonia Samia Khaddour, Emma Cristina Drăghici, Cosmin Mihai Mirițoiu, Ionela Elisabeta Staicu, Stelian Mihai Sever Petrescu, Răzvan Mercuț, Ionuț Cristian Resceanu, Alin Gabriel Ionescu, Sanda Mihaela Popescu

**Affiliations:** 1 Department of Oral Rehabilitation, University of Medicine and Pharmacy of Craiova, 200349 Craiova, Romaniasanda.popescu@umfcv.ro (S.M.P.); 2 Department of Applied Mechanics and Civil Constructions, Faculty of Mechanics, University of Craiova, 200585 Craiova, Romania; 3 Department of Orthodontics, University of Medicine and Pharmacy of Craiova, 200349 Craiova, Romania; 4 Department of Plastic and Reconstructive Surgery, University of Medicine and Pharmacy of Craiova, 200349 Craiova, Romania; 5 Department of Mechatronics and Robotics, Faculty de Automation, Computers, and Electronica, University of Craiova, 200440 Craiova, Romania; 6 Department of History of Medicine, Faculty of Medicine, University of Medicine and Pharmacy of Craiova, 200349 Craiova, Romania

**Keywords:** edentulous mouth, mouth rehabilitation, orthodontic treatment, aligner appliance, Dental Aesthetics Index (DAI), integrated care

## Abstract

**Background/Objectives**: The aim of the study was to determine the prevalence of molar absence and the need for limited preprosthetic orthodontic treatment in the situation of lateral molar edentulism in adult patients. **Methods**: The need for orthodontic treatment was studied retrospectively in a cohort of adult patients of both genders who presented for dental treatment at the Oral Rehabilitation Clinic of the University of Medicine and Pharmacy of Craiova. After applying the inclusion/exclusion criteria, the final study group consisted of 478 patients. The collected data were centralized in Microsoft Excel and statistical analyses were performed using SPSS software version 26. **Results**: The results of the study showed that more than 50% of adult patients need orthodontic treatment. The patients with the greatest need for orthodontic treatment also had a higher DMFT (*p* < 0.0005), indicating a higher need for dental caries treatment, as well as prosthetics of edentulous areas. The prevalence of molar edentulism was 57.31%. The acceptance of the treatment plan was inversely proportional to the need for orthodontic treatment. Tooth wear (*p* < 0.0005) and maxillary edentulism (*p* = 0.002) significantly contributed to a higher DAI. **Conclusions**: The need for orthodontic treatment increases with age, but the acceptance of oral rehabilitation plans that include orthodontic treatment is low. Better interdisciplinary collaboration is needed to ensure that adult patients benefit from the best treatment options.

## 1. Introduction

With the evolution of dentistry and recent developments in the field of orthodontics, patients’ esthetic demands have increased and thus the demand for orthodontic treatment among adult patients has increased [1,2,3,4]. The demand for orthodontic treatment by adults has increased significantly after the COVID-19 pandemic due to the so-called “Zoom boom” effect, whereby patients have become more aware of the appearance of their teeth due to working from home via the Zoom platform [5,6].

Orthodontic treatment for adult patients is usually targeted towards the age group between 20 and 30 years, but the notion of adult patients has gradually changed to include middle-aged and elderly patients, with the main reasons being the desire of patients to maintain natural teeth for as long as possible and to improve the functions of the stomatognathic system [7,8]. Some of the causes of the need for orthodontic treatment in adult patients are malocclusions untreated at the appropriate age, tooth loss with the absence of prosthetic rehabilitation, tooth migration following the loss of periodontal support, occlusal changes caused by inadequate dental therapies and tooth wear [9].

The need for preprosthetic orthodontic treatment is required in some cases to optimize both the esthetic and functional aspects of the masticatory system [10]. Pathological tooth migration as a result of edentulism is one of the clinical situations in which orthodontic treatment is indicated in adults to straighten teeth for prosthetic purposes. Pathological tooth migration has been defined as the change in tooth position resulting from the disruption of the forces that maintain the normal position of the teeth relative to the skull. The clinical manifestation of this condition is represented by the appearance of diastema, rotation, extrusion and proclination [11]. The first and second molars represent the teeth with the most pathologies causing extractions and lateral single-tooth edentulism, respectively. Following the extraction of a molar, the teeth adjacent to the edentulous gap move towards the edentulous space, either corporeally, by translation, closing the space, when the extraction is performed at a young age, or by tilting, producing a reduction only of the potential prosthetic space [12]. In adulthood, when the patient wishes to close the edentulous gap, treatment options include either prosthetics, which can only be done when the position of the adjacent teeth allows it, or the insertion of an implant after recreating the surgical and potentially prosthetic space through orthodontic treatment [12]. From a prosthetic point of view, preprosthetic othodontic treatment has the advantage of correctly positioning the teeth in order to facilitate the morphology of prosthetic restorations [9,13], allowing for minimally invasive prosthetic treatment [14].

The interdisciplinary approach combining restorative dentistry and orthodontics has proven to be particularly useful for the management of complex oral rehabilitation cases [15]. Bruxism is another situation in which orthodontic treatment with aligners can be useful. Characterized by involuntary grinding or clenching of the teeth, bruxism is a cause of tooth wear [16]. The challenges that may be encountered in patients with generalized tooth wear include malocclusion, missing teeth, loss of vertical occlusal dimension, impaired esthetic function, and potential prosthetic space affected by migration of adjacent teeth [17]. When aligners are used in patients with bruxism, they can also serve as a bruxism treatment appliance, which reduces the number of bruxism episodes and muscle hyperactivity [16,18,19].

For adult patients, the main motivational factor for accepting orthodontic treatment is the desire to improve esthetics, so the treatment of this category of patients is delicate, requiring a careful interdisciplinary approach [20,21,22,23]. The American Association of Orthodontics draws attention to the fact that orthodontic treatment in adults can, in addition to being an important source of income for orthodontists, confer a high risk of malpractice, because adults have, in addition to malocclusions requiring orthodontic treatment and systemic diseases, drug treatment and poor habits that can compromise the outcome of the treatment and can lead to complications that are difficult to treat [24].

From the recommendations made by Kokich in 2005 regarding the “state of the art” in adult orthodontics to the new digital techniques for individualized treatment recommended by the American Association of Orthodontics on their website, adult orthodontics has evolved greatly in the last 20 years [25].

The most common orthodontic treatment method is represented by fixed appliances, and the alternative option is represented by treatment with transparent thermoplastic aligners [1,2,26]. In some cases, patients who need orthodontic treatment refuse classic fixed appliances due to the difficulty of maintaining hygiene and chewing [27,28]. The literature has shown that fixed appliances and clear aligners have similar effects in terms of correcting tooth rotations, marginal ridge height, closing gaps and diastemas, and dental alignment, but fixed appliances are superior due to their advantage of correcting occlusal contacts [29,30,31,32]. Also, some studies in the literature have reported more frequent relapses in patients who underwent treatment with clear aligners than in those who underwent treatment with fixed appliances [32].

Establishing the need for orthodontic treatment in adults as clearly as possible is very important to avoid malpractice and overtreatment. The need for orthodontic treatment is defined as the need for orthodontic intervention assessed by the orthodontic specialist doctor, and the request for orthodontic treatment is defined as a subjective desire self-perceived by the patient, usually for esthetic or social reasons [33]. There are many factors that must be taken into account before the development of an orthodontic treatment plan. Thus, to assess the objective need for treatment, a series of indices have been developed, such as the index of orthodontic treatment need (IOTN), the index of complexity, outcome and need for orthodontic treatment (ICON), the index of dental esthetics (DAI), the index of orthodontic treatment priority (TPI) or the inter-par evaluation index (PAR) [32,33,34,35,36]. The dental esthetic index (DAI) was introduced in 1986 and uses objective and subjective clinical esthetic factors to measure the severity of the problem and the need for orthodontic treatment [37]. It comprises 10 occlusal elements that measure the need for orthodontic treatment, and the final results can be classified into four levels of malocclusion, from minor to severe [34,37].

The motivation for the present study arose from the increase in requests for orthodontic treatment in adult patients in general dentistry offices in the Oltenia area. Molar absence is among the most common types of edentulism, which occurs when a six-year molar is lost at an early age. The study aimed to determine the prevalence of lateral molar edentulism and the need for limited preprosthetic orthodontic treatment in the situation of lateral single-tooth edentulism in adult patients in the Oltenia region, Romania.

## 2. Materials and Methods

### 2.1. Study Design

This retrospective study included a total of 478 patients who presented themselves to the Oral Rehabilitation Department of the University of Medicine and Pharmacy of Craiova for the rehabilitation of edentulous spaces following the extraction of the first or second maxillary or mandibular molar. The data collection and analysis were performed over a period of one year (January 2025–January 2026) and involved the evaluation of 834 dental charts from the Oral Rehabilitation Discipline database, collected over a 10-year period (2013–2023). The data taken from the medical records were: gender, age, residence, DMFT index, the number of single lateral molar edentulism, the number of patients for whom pre-prosthetic orthodontic treatment was proposed, and the number of patients who accepted pre-prosthetic orthodontic treatment. Observation records, panoramic radiographs or digital orthopantomography, study models, and the discipline’s intraoral photography archive were used to obtain these data. The study protocol was approved by the Ethics Committee of the University of Medicine and Pharmacy of Craiova (Approval Number 156, 30 August 2023).

### 2.2. Patient Selection

The charts of the patients selected for inclusion in the study met the inclusion/exclusion criteria. After applying the inclusion/exclusion criteria, the final study group consisted of 478 patients. The minimum sample size was determined a priori using G*Power 3.1.9.7, Heinrich Heine University Düsseldorf, Germany [38,39], considering the statistical analysis tests to be performed (Chi-square test, Kruskal–Wallis test approximated as the F-family test), a medium effect size of 0.25, a two-sided significance level of *α* = 0.05, and a desired power 1-β equal to 0.95. Thus, the analysis indicated a minimum total sample size of 317 participants.

*Inclusion criteria:*

- Adult patients, over 18 years of age;
- Patients with single-tooth edentulism at the level of the first or second maxillary/mandibular molars;
- Patients with potential prosthetic space reduced by migration of adjacent teeth;
- Patients with good systemic health, classified in ASA I and ASA II classes;
- Patients with satisfactory oral hygiene;
- Compliant patients.

*Exclusion criteria:*

- Patients under 18 years of age;
- Adult patients with multiple, extensive, total edentulism, treated or untreated;
- Patients with unsatisfactory oral hygiene;
- Patients with poor health, with systemic conditions that determine classification in a higher ASA class than ASA II.

### 2.3. Calculating the Dental Esthetics Index DAI

To evaluate the dental esthetics index, DAI, several elements quantified with a value were used [34,40], according to Table 1.

The values that allow quantifying the need for orthodontic treatment according to the DAI are presented in Table 2. The DAI value was obtained by summing the values of the parameters evaluated according to Table 1. Thus, the following 4 levels of malocclusion were used: DAI 1 (≤25), with normal occlusion and no need for orthodontic treatment, DAI 2 (26–30), with mild malocclusion and elective recommendation for orthodontic treatment, DAI 3 (31–35) with moderate malocclusion and recommended orthodontic treatment, and DAI 4 (≥36) with severe malocclusion and necessary orthodontic treatment.

### 2.4. Variables

The variables studied were:

- Demographic elements: gender, age, residence;
- DMFT index;
- Presence of tooth wear;
- Presence of bruxism;
- Presence of periodontal disease;
- Presence of crowded teeth;
- Number of patients for whom pre-prosthetic orthodontic treatment was proposed;
- Presence of tooth migration;
- Presence of occlusal dysfunction;
- Acceptance of orthodontic treatment: number of patients who accepted pre-prosthetic orthodontic treatment.

### 2.5. Statistical Analysis

The collected data were centralized in Microsoft Excel (Microsoft Corporation, Redmond, WA, USA). Descriptive statistics were expressed as mean ± standard deviation (SD) or median values for continuous variables and as frequency distributions with associated percentages for categorical and ordinal parameters. Statistical analyses were performed using SPSS software, version 26 (IBM Corp., Armonk, NY, USA). Continuous parameters were assessed for normality using the Shapiro–Wilk test. Due to the ordinal and non-normally distributed nature of clinical indices, non-parametric methods were applied. Comparisons between multiple independent groups were performed using the Kruskal–Wallis H test. Associations between categorical variables were evaluated using the Chi-square test (χ^2^). The influence of age, DMFT index, presence of clinical manifestations regarding teeth migration, tooth wear, bruxism, periodontal disease, temporomandibular joint disfunction, and maxillary or mandibular edentation on the DAI was assessed through a cumulative-odds ordinal logistic regression, with proportional odds. The level of statistical significance was set at *p* < 0.05.

## 3. Results

The need for orthodontic treatment was studied retrospectively in a cohort of patients aged between 18 and 65 years, of both genders, who presented for dental treatment in the Department of Oral Rehabilitation of the University of Medicine and Pharmacy of Craiova, over a 10-year period (2013–2023). In total, 834 dental charts were evaluated and only files of patients with molar edentulism were included in the study, representing 57.31% of all patients registered for treatment in the studied period. Table 3 presents the basic characteristics of the studied cohort. A total of 478 patients were included in the study, of which 55.4% were women and 44.6% were men, aged between 18 and 65 years, with a mean age of 30.3 ± 11.8 years. Of these, 50% were aged between 18 and 25 years, 30.25% were aged between 26 and 40 years and 19.5% were aged between 41 and 65 years. The majority of patients (85.8%) came from urban areas. The DMFT index of the cohort was on average 9.5 ± 5.7. The DAI described above was used to assess the need for orthodontic treatment (Table 3).

The statistical analysis followed the distribution of DAI according to gender, age, age groups, residence and DMFT index. The analysis showed that over 50% of patients needed orthodontic treatment, 43.5% of them being recommended orthodontic treatment (DAI 3) and 9.8% having an indication of necessary orthodontic treatment (DAI 4). The distribution of DAI according to gender was similar, the majority having a DAI of 3. The distribution of DAI according to age showed that those with DAI 4 had a mean age of 41.3 ± 13.5 and a median of 41, significantly higher than the other groups, with lower DAI values, with a median age between 25 and 29 years. The distribution of patients according to the DAI 2 and DAI 3 indices was similar, with the majority being from the 18–25 age group, followed by those in the 26–40 age group and the fewest from the 41–65 age group. On the contrary, the majority of those with DAI 4, i.e., necessary orthodontic treatment, came from the 41–65 age group (51.1% respectively), followed by those in the 26–40 age group (31.9% respectively), the fewest being in the 18–25 age group. This difference in the distribution of DAI between age groups was statistically significant. Thus, as patients get older, malocclusion is associated with more severe orthodontic treatment needs. The distribution of DAI according to residence did not show significant differences. The distribution of DMFT value according to DAI showed that those with DAI 3 and 4 had DMFT 10, significantly higher than those with DAI 1 and 2 who had DMFT 7. Thus, those with greater needs for orthodontic treatment also have greater needs for dental caries treatment (Table 3).

Patients with tooth migration have a high percentage of need for orthodontic treatment, which is statistically significant (*p* < 0.0005) (Table 4). The prevalence of lateral molar edentulism was not statistically significantly different between the four patient subgroups, but was much more common at the maxillary level.

The gender distribution was homogeneous. There were no statistically significant differences between genders in terms of the type of lateral molar edentulism, the presence of dentoalveolar incongruence, the presence of tooth migration, the presence of attrition tooth wear, the presence of bruxism, the presence of periodontal disease, the types of DAI and the degree of acceptance of orthodontic treatment (Table 5).

The statistical analysis of the investigated variables according to age groups highlights several relevant aspects; the distribution of the type of lateral molar edentulism did not present statistically significant differences between age groups. Maxillary lateral molar edentulism was predominant in all analyzed groups, representing 72.4% in the 18–25-year age group, 67.8% in the 26–40-year age group and 67.7% in the 41–65-year age group. Mandibular lateral molar edentulism was less frequent. The presence of dentoalveolar incongruence was relatively similar in all age groups, without statistically significant differences (*p* = 0.637). Tooth migrations presented statistically significant differences between age groups (*p* < 0.0005). These were very common in the 18–25-year age group (95.8%), but the frequency decreased in the older age groups (83.6% and 81.7%). Attrition-type tooth wear did not show statistically significant differences between the analyzed groups (*p* = 0.368). Its prevalence was relatively low, ranging between 16.3% and 22.6%. Bruxism showed statistically significant differences between age groups (*p* = 0.003). The proportion of patients with bruxism was significantly higher in the 41–65-year age group (31.2%) compared to younger groups (16.3% and 15.8%). Regarding the presence of periodontal disease, it did not show statistically significant differences between age groups (*p* = 0.176). However, a trend of decreasing prevalence with age is observed within the analyzed sample (Table 6).

The distribution of DAI scores showed highly statistically significant differences between age groups. Severe malocclusion (DAI category 4) was much more common in the 41–65-year age group (25.8%) compared to younger groups. Regarding acceptance of the orthodontic treatment plan, no statistically significant differences were identified between age groups (*p* = 0.115), with most patients refusing orthodontic treatment (Table 6).

The analysis of variables according to the presence or absence of occlusal dysfunction highlights several statistically relevant associations. The distribution of the type of lateral molar edentulism did not show statistically significant differences between patients with occlusal dysfunction and those without occlusal dysfunction (*p* = 0.61). Mandibular lateral molar edentulism was more frequent in the group with occlusal dysfunction (63.46%) compared to maxillary lateral molar edentulism (36.54%). The majority of patients with occlusal dysfunction presented dentoalveolar incongruence (94.9%). Tooth migrations were frequent in both groups: 94.9% in patients with occlusal dysfunction and 88.2% in those without, but this did not show a statistically significant association with occlusal dysfunction (*p* = 0.117). Attrition-type tooth wear showed a highly statistically significant association with occlusal dysfunction (*p* < 0.0005). It was present in 68.4% of patients with occlusal dysfunction, compared to only 7.5% of those without dysfunction. Bruxism also showed a significant association with occlusal dysfunction (*p* < 0.0005). The proportion of patients with bruxism was much higher in the occlusal dysfunction group (72.2%) compared to the group without dysfunction (8.5%). The presence of periodontal disease did not show statistically significant differences between the two groups (*p* = 0.372), being relatively similarly distributed (Table 7).

The distribution of DAI scores did not show statistically significant differences between patients with and without occlusal dysfunction (*p* = 0.392). The majority of patients fell into the DAI 2 and DAI 3 categories. No statistically significant differences were observed in terms of acceptance of the orthodontic treatment plan between the two groups (*p* = 0.611). In both groups, the majority of patients did not accept the treatment (Table 7).

A cumulative-odds ordinal logistic regression with proportional odds was run to determine the effect of age, DMFT index, and the presence of clinical manifestations (teeth migration, tooth wear, bruxism, periodontal disease, temporomandibular joint disfunction, maxillary or mandibular edentation) on the DAI. There were proportional odds, as assessed by a full likelihood-ratio test comparing the fitted model to a model with varying location parameters, χ^2^(16) = 35.711, *p* = 0.077. The deviance goodness-of-fit test indicated that the model was a good fit to the observed data, χ^2^(1216) = 806.524, *p* = 0.989, but most cells were sparse with zero frequencies in 72.9% of cells. However, the final model statistically significantly predicted the DAI over and above the intercept-only model, χ^2^(8) = 239.192, *p* < 0.0005.

The odds of patients with tooth wear having a higher DAI was 143.51 times higher than for patients without tooth wear, (95% CI, 24.121 to 853.815), χ^2^(1) = 29.794, *p* < 0.0005. Also, the odds of patients with maxillary edentulism having a higher DAI was 1.945 times higher than that for patients with mandibular edentulism, (95% CI, 1.278 to 2.96), χ^2^(1) = 9.636, *p* = 0.002.

On the other hand, the odds of patients with teeth migration having a higher DAI was 0.017 times less than for patients without teeth migration, (95% CI, 0.008 to 0.038), χ^2^(1) = 99.601, *p* < 0.0005. Similar results are obtained for patients with bruxism, with the odds being 0.02 times less than for patients without bruxism, (95% CI, 0.004 to 0.112), χ^2^(1) = 19.833, *p* < 0.0005, patients with periodontal disease, with the odds being 0.313 times less than for patients without periodontal disease, (95% CI, 0.200 to 0.490), χ^2^(1) = 25.768, *p* < 0.0005, and patients with TMJ disfunction, with the odds being 0.070 times less than for patients without TMJ disfunction, (95% CI, 0.035 to 0.141), χ^2^(1) = 56.352, *p* < 0.0005.

An increase in age or DMFT index was not associated with an increase in the odds of having a higher DAI, *p* = 0.0180 for age and *p* = 0.622 for DMFT.

Overall, tooth wear and maxillary edentulism significantly contribute to a higher DAI.

## 4. Discussion

The present study revealed that the prevalence of molar edentulism is over 50% in the studied adult population. Also, in a recent Bulgarian study, a similar percent of molar loss was reported [41]. The literature has shown that factors such as gender, age, background or sociodemographic factors can influence quality of life related to oral health [27,42]. The field of orthodontics has evolved significantly in recent years, and the demand for orthodontic treatment has also increased among adult patients [43,44]. In Romania, there has also been an increase in the demand for orthodontic services by adult patients after the COVID 19 pandemic. Adult patients seek orthodontic treatment to improve dental esthetics, oral functionality and psychosocial well-being [44,45,46]. Orthodontic services for adult patients are also beginning to be more requested by doctors who practice implantology in order to create the optimal space for dental implants.

Tooth wear and maxillary molar edentulism had a significant contribution to a higher DAI. Since DAI is an esthetic evaluation index, the association with tooth wear and maxillary edentulism is explained by itself, as they both affect oral esthetics. Among the indices used to assess the need for orthodontic treatment, the DAI has been considered reliable, with good sensitivity, and therefore appropriate for epidemiological studies, although it takes longer to calculate compared to other indices [47]. Orthodontic treatment need assessment indices provide information about the distribution of population need and treatment priority [37]. The dental esthetics index (DAI) assesses the relative social acceptability of dental appearance based on the general understanding of dental esthetics. This index is mainly used to identify the need for orthodontic treatment and as a screening tool to determine treatment priority. Although the DAI has advantages, such as simplicity, reliability and easy reproducibility of data, it also has limitations such as not including cases with crossbite, posterior open bite, midline deviation or covered overbite [48]. In the study conducted by Golfeshan et al., the DAI and OI indices were used to assess the need for orthodontic treatment in a group of students at the Faculty of Dentistry in Shiraz. The results of their study showed that the need for orthodontic treatment based on the DAI and the OI index was higher than the demand for orthodontic treatment, although the study was conducted on a population aware of orthodontic treatments and the risk of not performing them [37]. In the present study, the need for orthodontic treatment was also high, and the demand, i.e., the rate of acceptance of the orthodontic treatment plan, was reduced, with most patients refusing orthodontic treatment. An important aspect is the psychological impact of orthodontic treatment on adult patients, who feel embarrassed by wearing dental braces [49,50]. In the study conducted by Pattanaik et al. a significant negative correlation was found between age and acceptance of orthodontic treatment, with younger patients being more adherent to this type of treatment [44].

The present study showed that over 50% of the patients needed orthodontic treatment. The analysis showed that the need for orthodontic treatment increases as patients age, and malocclusion evolves and becomes more severe. A study conducted on 639 Mexican patients of all ages (7–62 years) aimed to evaluate the prevalence of the need for orthodontic treatment based on the DAI, taking into account factors such as gender and age [51]. The results of the study confirm those of other previous studies that highlight the role of occlusion disorders such as open occlusion and dentoalveolar incongruence in calculating a DAI of severe malocclusion. Although over 50% of the female participants in the study and over 40% of the male participants in the study had DAI indices of 3 or 4, the associations of the need for orthodontic treatment with the patients’ gender or age were not significant. However, the high prevalence of severe malocclusion emphasizes the need for early orthodontic intervention in Mexican populations [50,51].

The results of the present study showed that patients with the greatest need for orthodontic treatment also have the greatest need for dental caries treatment, with a high DMFT. This result correlates with the results obtained in other population studies, such as the study conducted by a team of researchers from a dental school in Paraguay in 2017 which was also on a group of adult patients. It is worth noting that the indices obtained by us in this study are similar to what was obtained in this study by the Paraguayan researchers [52]. It is certain that the two indices correlate, especially in populations where although there is high availability for dental services with the number of dentists being very high, the amount spent on the prevention of oral diseases is very small, and the vast majority of the population pays for dental services in full [53].

Occlusal dysfunction is statistically significantly associated with dentoalveolar incongruence, attrition-type tooth wear, and bruxism. This is not the first study conducted in Romania to show this; bruxism and attrition-type tooth wear is correlated with occlusal dysfunction in both young and elderly adults [54,55].

Because all patients included in the study presented with single-tooth molar edentulism, in addition to the need for treatment of dental caries, tooth wear, and management of bruxism, they also need prosthetics or another type of edentulism resolution, such as mesialization of the molars in place of edentulism [56]. The interdisciplinary approach to clinical cases offers the advantage of a thorough examination and individualized planning adapted to the patients’ requirements [57,58,59]. There are numerous studies in the literature in which orthodontic treatment has been shown to be beneficial before prosthetic treatment, obtaining optimal clinical results. However, the interdisciplinary approach also presents disadvantages such as financial and time considerations, and a detailed explanation of the procedures and treatment objectives is necessary to motivate patients [60].

Patient satisfaction is a critical factor in the success of dental treatment, so dentists should clearly explain the treatment process, provide realistic expectations, and establish a well-defined treatment plan from the outset [61]. Thus, the choice of treatment should be tailored to the clinical need and requirements in order to achieve optimal clinical results.

The study conducted by Dayanand et al. aimed to assess the awareness, motivation and willingness of patients to undergo orthodontic treatment, observing whether age is perceived as a barrier, and their results showed that patients of all ages recognize the benefits of orthodontic treatment for improving dental health [62]. In addition, the study conducted by Sandhya et al. showed that the number of adult patients accepting orthodontic treatment exceeded the number of children or adolescents [40].

Several studies in the literature have compared the treatment outcomes of fixed classic braces with those of clear aligners, and their conclusions have shown that although aligners can provide superior esthetics, increased comfort, and better oral hygiene, they may have limitations in controlling certain tooth movements [50,63,64,65]. Controlling tooth tilt is a challenge for clear aligners, especially in cases requiring tilt adjustment greater than 3 degrees [26]. The study by Pham et al. showed that clear aligners may benefit from shorter treatment times and higher patient satisfaction, but fixed classic braces offer superior control over tooth movement [26].

A limitation of the study is that the research was conducted in a single center, with demographic homogeneity, which does not reflect a broader patient population; thus, future research focusing on a more diverse patient population is needed to improve the generalizability of the findings. Although the Oltenia region has approximately a 40% rural population, their access to dental services is low, especially for orthodontic treatments, hence the high proportion of patients from the urban population in the study [66]. However, pre-prosthetic orthodontic treatment is part of the current interdisciplinary trend to improve prosthetic conditions. The arguments are esthetic, biological, biomechanical and prophylactic. Thus, the need for orthodontic treatment for adults will form a subject for new research in the future.

## 5. Conclusions

The prevalence of molar edentulism was higher than 50% (57.31%). Most adult patients with molar edentulism need orthodontic treatment. Most adult patients included in the study need orthodontic treatment. The need for orthodontic treatment increases with age, as malocclusion evolves and becomes more severe. Although the need for orthodontic treatment is high, the demand for orthodontic treatment is low and the acceptance of oral rehabilitation plans that include orthodontic treatment is even lower. Patients with the greatest need for orthodontic treatment also have the greatest need for treatment of dental caries, as well as treatment of edentulism and its complications. Both tooth wear and maxillary edentulism have a significant contribution to a higher DAI. Better collaboration between general dentists and orthodontists is necessary so that adult patients benefit from advantageous rehabilitation that preserves as many natural teeth as possible in the arch by including orthodontics in the complex oral rehabilitation treatment plan.

## Figures and Tables

**Table 1:** Determination of the DAI [34,40].

DAI	Overjet	Overbite	Open bite	Missing Incisors	Anterior Dental Crowding	Anterior Dental Spacing	Diastema	Interincisal Line Deviation
	10	5	3	10	3	2	1	3

**Table 2:** DAI values [34,40].

DAI	Severity	Indication
≤25	Normal occlusion	No treatment required
26–30	Mild	Elective treatment
31–35	Moderate	Recommended treatment
≥36	Severe	Orthodontic treatment required

**Table 3:** Distribution of the study group according to the DAI.

Variables	Category	Subgroup				Total	*p*
		DAI 1	DAI 2	DAI 3	DAI 4		
Patients (no.)		56 (11.7%)	167 (34.9%)	208 (43.5%)	47 (9.8%)	478 (100%)	
Gender	F	35 (7.3%)	98 (20.5%)	108 (22.6%)	24 (5%)	265 (55.4%)	0.355 *
		35 (62.5%)	98 (58.7%)	108 (51.9%)	24 (51.1%)		
	M	21 (4.4%)	69 (14.4%)	100 (20.9%)	23 (4.8%)	213 (44.6%)	
		21 (37.5%)	69 (41.3%)	100 (48.1%)	23 (48.9%)		
Age (years)	Median	29	25	25	41	26	<0.0005 **^†^
	Mean ± SD	35.8 ± 15.1	29.1 ± 10	29.3 ± 10.9	41.3 ± 13.5	30.3 ± 11.8	
Age groups (years)	18–25	16 (3.3%)	96 (20.1%)	119 (24.9%)	8 (1.7%)	239 (50%)	<0.0005 *^†^
		16 (28.6%)	96 (57.5%)	119 (57.2%)	8 (17%)		
	26–40	22 (4.6%)	49 (10.3%)	60 (12.6%)	15 (3.1%)	146 (30.5%)	
		22 (39.3%)	49 (29.3%)	60 (28.8%)	15 (31.9%)		
	41–65	18 (3.8%)	22 (4.6%)	29 (6.1%)	24 (5%)	93 (19.5%)	
		18 (32.1%)	22 (13.2%)	29 (13.9%)	24 (51.1%)		
Residence	Urban	46 (9.6%)	142 (29.7%)	180 (37.7%)	42 (8.8%)	410 (85.8%)	0.735 *
		46 (82.1%)	142 (85%)	180 (86.5%)	42 (89.4%)		
	Rural	10 (2.1%)	25 (5.2%)	28 (5.9%)	5 (1%)	68 (14.2%)	
		10 (17.9%)	25 (15%)	28 (13.5%)	5 (10.6%)		
DMFT index	Median	7	7	10	10	9	<0.0005 **^†^
	Mean ± SD	8.2 ± 5.8	8.3 ± 5.4	10.6 ± 5.7	10.3 ± 5.5	9.5 ± 5.7	

* Chi-square test. ** Kruskal–Wallis H test. ^†^ Statistically significant. The values in gray are summed by columns, representing the distribution of patients of different age groups, according to each parameter described in a line. The column total contains the distribution by age group for each category of the parameters described in a line.

**Table 4:** Distribution of studied variables according to the need for orthodontic treatment.

Variables	Category	Subgroup				Total	*p*
		DAI 1	DAI 2	DAI 3	DAI 4		
Patients (no.)		56 (11.7%)	167 (34.9%)	208 (43.5%)	47 (9.8%)	478	
Lateral molar edentulism type	Maxilla	40 (71.4%)	114 (68.3%)	143 (68.8%)	38 (80.9%)	335	0.547 *
	Mandible	16 (28.6%)	53 (31.7%)	65 (31.2%)	9 (19.1%)	143	
Dentoalveolar incongruence	Present	30 (53.6%)	87 (52.1%)	113 (54.3%)	27 (57.4%)	257	0.637 *
	Absent	26 (46.4%)	80 (47.9%)	95 (45.7%)	20 (42.6%)	221	
Dental migrations	Present	52 (92.9%)	140 (83.8%)	185 (88.9%)	44 (93.6%)	427	<0.0005 *^†^
	Absent	4 (7.1%)	27 (16.2%)	23 (11.1%)	3 (6.4%)	51	
Attrition tooth wear	Present	9 (16.1%)	27 (16.2%)	37 (17.8%)	11 (23.4%)	84	0.368 *
	Absent	47 (83.9%)	140 (83.8%)	171 (82.2%)	36 (76.6%)	394	
Bruxism	Present	9 (16.1%)	26 (15.6%)	41 (19.7%)	15 (31.9%)	91	0.003 *^†^
	Absent	47 (83.9%)	141 (84.4%)	167 (80.3%)	32 (68.1%)	387	
Periodontal disease	Present	20 (35.7%)	47 (28.1%)	67 (32.2%)	18 (38.3%)	152	0.176 *
	Absent	36 (64.3%)	120 (71.9%)	141 (67.8%)	29 (61.7%)	326	
Acceptance of orthodontic treatment plan	Yes	4 (7.1%)	11 (6.6%)	13 (6.3%)	5 (10.6%)	33	0.115 **
	No	52 (92.9%)	156 (93.4%)	195 (93.7%)	42 (89.4%)	445	

* Chi-square test. ** Chi-square test with Monte Carlo simulation. ^†^ Statistically significant.

**Table 5:** Distribution of studied variables by gender.

Variables	Category	Subgroup		Total	*p*
		Gender			
		Female	Male		
Lateral molar edentulism type	Maxilla	192 (72.45%)	143 (67.14%)	335	0.207 *
	Mandible	73 (27.55%)	70 (32.86%)	143	
Dentoalveolar incongruence	Present	134 (50.6%)	123 (57.7%)	257	0.14 *
	Absent	131 (49.4%)	90 (42.3%)	221	
Dental migrations	Present	236 (89.1%)	191 (89.7%)	427	0.95 *
	Absent	29 (10.9%)	22 (10.3%)	51	
Attrition tooth wear	Present	46 (17.4%)	38 (17.8%)	84	0.99 *
	Absent	219 (82.6%)	175 (82.2%)	394	
Bruxism	Present	50 (18.9%)	41 (19.2%)	91	1 *
	Absent	215 (81.1%)	172 (80.8%)	387	
Periodontal disease	Present	82 (30.9%)	66 (31.0%)	148	1 *
	Absent	183 (69.1%)	147 (69.0%)	330	
DAI	1	35 (13.2%)	21 (9.9%)	56	0.36 *
	2	98 (37.0%)	69 (32.4%)	167	
	3	108 (40.8%)	100 (46.9%)	208	
	4	24 (9.1%)	23 (10.8%)	47	
Acceptance of orthodontic treatment plan	Yes	16 (6.0%)	17 (8.0%)	33	0.52 *
	No	249 (94.0%)	196 (92.0%)	445	

* Chi-square test.

**Table 6:** Distribution of studied variables according to age groups.

Variables	Category	Age groups			Total	*p*
		18–25	26–40	41–65		
Lateral molar edentulism type	Maxilla	173 (72.4%)	99 (67.8%)	63 (67.7%)	335	0.547 *
	Mandible	66 (27.6%)	47 (32.2%)	30 (32.3%)	143	
Dentoalveolar incongruence	Present	127 (53.1%)	76 (52.1%)	54 (58.1%)	257	0.637 *
	Absent	112 (46.9%)	70 (47.9%)	39 (41.9%)	221	
Dental migrations	Present	229 (95.8%)	122 (83.6%)	76 (81.7%)	427	<0.0005 *^†^
	Absent	10 (4.2%)	24 (16.4%)	17 (18.3%)	51	
Attrition tooth wear	Present	39 (16.3%)	24 (16.4%)	21 (22.6%)	84	0.368 *
	Absent	200 (83.7%)	122 (83.6%)	72 (77.4%)	394	
Bruxism	Present	39 (16.3%)	23 (15.8%)	29 (31.2%)	91	0.003 *^†^
	Absent	200 (83.7%)	123 (84.2%)	64 (68.8%)	387	
Periodontal disease	Present	85 (35.6%)	43 (29.5%)	24 (25.8%)	152	0.176 *
	Absent	154 (64.4%)	103 (70.5%)	69 (74.2%)	326	
DAI	1	16 (6.7%)	22 (15.1%)	18 (19.4%)	56	<0.0005 *^†^
	2	96 (40.2%)	49 (33.6%)	22 (23.7%)	167	
	3	119 (49.8%)	60 (41.1%)	29 (31.2%)	208	
	4	8 (3.3%)	15 (10.3%)	24 (25.8%)	47	
Acceptance of orthodontic treatment plan	Yes	18 (7.5%)	13 (8.9%)	2 (2.2%)	33	0.115 *
	**No**	221 (92.5%)	133 (91.1%)	91 (97.8%)	445	

* Chi-square test. ^†^ Statistically significant.

**Table 7:** Distribution of the studied variables according to the presence of occlusal dysfunction.

Variables	Category	Subgroup		Total	*p*
		With Occlusal Dysfunction	Without Occlusal Dysfunction		
Lateral molar edentulism type	Maxilla	33 (63.46%)	302 (70.89%)	335	0.269 *
	Mandible	19 (36.54%)	124 (29.11%)	143	
Dentoalveolar incongruence	Present	75 (94.9%)	182 (45.6%)	257	<0.0005 *^†^
	Absent	4 (5.1%)	217 (54.4%)	221	
Dental migrations	Present	75 (94.9%)	352 (88.2%)	427	0.117 *
	Absent	4 (5.1%)	47 (11.8%)	51	
Attrition tooth wear	Present	54 (68.4%)	30 (7.5%)	84	<0.0005 *^†^
	Absent	25 (31.6%)	369 (92.5%)	394	
Bruxism	Present	57 (72.2%)	34 (8.5%)	91	<0.0005 *^†^
	Absent	22 (27.8%)	365 (91.5%)	387	
Periodontal disease	Present	29 (36.7%)	123 (30.8%)	152	0.372 *
	Absent	50 (63.3%)	276 (69.2%)	326	
DAI	1	6 (7.6%)	50 (12.5%)	56	0.392 *
	2	28 (35.4%)	139 (34.8%)	167	
	3	34 (43.0%)	174 (43.6%)	208	
	4	11 (13.9%)	36 (9.0%)	47	
Acceptance of orthodontic treatment plan	Yes	7 (8.9%)	26 (6.5%)	33	0.611 *
	No	72 (91.1%)	373 (93.5%)	445	

* Chi-square test. ^†^ Statistically significant.

## Data Availability

The authors declare that the data of this research are available from the corresponding authors upon reasonable request.

## Abbreviations

The following abbreviations are used in this manuscript:

IOTN	Index of Orthodontic Treatment Need
ICON	Index of Complexity, Outcome and Need for Orthodontic Treatment
DAI	Dental Esthetic Index
TPI	Orthodontic Treatment Priority Index
PAR	Peer Assessment Rating Index
DMFT	Decayed, Missing, and Filled Teeth Index
ASA	American Society of Anesthesiologists
SD	Standard Deviation
SPSS	Statistical Package for the Social Sciences
OI	Occlusal Index
